# Cyperotundone combined with adriamycin induces apoptosis in MCF-7 and MCF-7/ADR cancer cells by ROS generation and NRF2/ARE signaling pathway

**DOI:** 10.1038/s41598-022-26767-x

**Published:** 2023-01-25

**Authors:** Wenna Shao, Xinzhao Wang, Zhaoyun Liu, Xiang Song, Fukai Wang, Xiaoyu Liu, Zhiyong Yu

**Affiliations:** 1 First Clinical College, Shandong University of Traditional Chinese Medicine, Jinan, 250014 Shandong People’s Republic of China; 2 Breast Cancer CenterShandong Cancer Hospital and Institute, Shandong Academy of Medical Sciences, Shandong First Medical University, Jinan, 250117 Shandong People’s Republic of China; 3 RemeGen, Ltd, 58 Middle Beijing Road, Yantai Economic & Technological Development Area, Yantai, 264006 Shandong People’s Republic of China

**Keywords:** Breast cancer, Apoptosis

## Abstract

Breast cancer has become the most prevalent cancer, globally. Adriamycin is a first-line chemotherapeutic agent, however, cancer cells acquire resistance to it, which is one of the most common causes of treatment failure. ROS and NRF2 are essential oxidative stress factors that play a key role in the oxidative stress process and are associated with cancer. Our goal is to create novel therapeutic drugs or chemical sensitizers that will improve chemotherapy sensitivity. The optimal concentration and duration for MCF-7 and MCF-7/ADR cells in ADR and CYT were determined using the CCK-8 assay. We found that ADR + CYT inhibited the activity of MCF-7 and MCF-7/ADR cells in breast cancer, as well as causing apoptosis in MCF-7 and MCF-7/ADR cells and blocking the cell cycle in the G0/G1 phase. ADR + CYT induces apoptosis in MCF-7 and MCF-7/ADR cells through ROS generation and the P62/NRF2/HO-1 signaling pathway. In breast cancer-bearing nude mice, ADR + CYT effectively suppressed tumor development in vivo. Overall, our findings showed that CYT in combination with ADR has potent anti-breast cancer cell activity both in vivo and in vitro, suggesting CYT as the main drug used to improve chemosensitivity.

## Introduction

According to the International Agency for Research on Cancer Survey statistics, breast cancer has become the most prevalent cancer in the world^1^. Chemotherapy is one of the most commonly used breast cancer therapies. However, some patients develop resistance to chemotherapy, which often results in treatment failure. Adriamycin is widely used as a first-line chemotherapeutic drug in solid and hematological malignancies, which invariably leads to drug resistance and poor prognosis^2,3^.As a result, it is important to investigate innovative therapeutic approaches to increase patients' susceptibility and prevent resistance to chemotherapeutic agents.

*Cyperus rotundus* L. is a kind of Chinese herbal medicine, that has been used in China to treat qi stagnation and blood stasis diseases for a long time, such as depression and dysmenorrhea^4^. At present, a few preclinical studies have shown that *Cyperus rotundus* L. extract can act as a cytotoxic agent, effectively killing all types of tumor cells such as breast cancer cell MCF-7, cervical cancer cell HeLa, hepatoma cell HepG2, prostate cancer cell (PC3), colorectal cancer cell (HT29), etc., demonstrating a broad spectrum of anti-cancer activity ^5,6^. Researchers extracted the anticancer active component 11,12-dihydroxy-4-ene-3-one from an ethyl acetate extract of *Cyperus rotundus* L. and found that it had a significant cytotoxic effect on ovarian cancer cells^6^. Our early findings also demonstrated the anticancer activity of *Cyperus rotundus* L. ethanol extract (EECR) on triple-negative breast cancer^7,8^.Cyperotundone (C_15_H_22_O), one of the monomers of *Cyperus rotundus* L., has been shown potent cytotoxic effect on breast cancer cells MCF-7 at a certain dose. However, there have been no studies on the impact and molecular mechanism of CYT + ADR in tumor progression, so we aimed to investigate the mechanism of CYT + ADR in MCF-7 and MCF-7/ADR cells.

Recently, oxidative stress (OS) has been found as a crucial component in chemoresistance regulation, although the mechanism still needs to be elucidated^9,10^. Reactive oxygen species (ROS) are oxygenates that are directly or indirectly converted from molecular oxygen and have a more chemical reactivity than molecular oxygen^11,12^. Under normal circumstances, ROS levels are kept within a certain range to maintain the oxidative and antioxidant systems in balance. With the in-depth investigation of the biological function of ROS in recent years, the lethal impact of ROS on tumor cells has steadily been discovered^13–15^. Moreover, an increase in ROS may push cancer cells past the life threshold, resulting in the activation of several cell death pathways thus, limiting cancer progression^16^. NRF2 is one of the important protective factors against oxidative stress. A large number of studies have shown that NRF2/ARE has a dual function in malignancies. On the one hand, activated NRF2/ARE pathways can enhance the body's antioxidation and detoxification, thereby preventing the development of tumors; on the other hand, overactivated NRF2 can decrease tumor cell apoptosis by minimizing the oxidative stress caused by tumors, thereby promoting tumor development and enhancing the resistance of tumor tissues to radiotherapy and chemotherapy^17–19^. As a result, it is important to continue investigating the activators and inhibitors of this pathway as potential targets for cancer-targeting drugs to develop a new therapeutic intervention for breast cancer.

P62 interacts with the NRF2 signaling pathway as a scaffold in the autophagy signaling pathway^20^. Inhibition of autophagy or other factors causes the accumulation of P62 in cells, which competes with Keap1 for binding, preventing NRF2 and Keap1 from binding, resulting in the accumulation of NRF2 in cells and promoting tumorigenesis. The activation of NRF2 results in the activation of HO-1 as well. Even though many aspects of the P62/NRF2/HO-1 signaling pathway and its interaction with ROS are unknown, studies have indicated that it is involved in several diseases, including tumor incidence, development, and therapy^20^. Further research into the complicated regulatory mechanism of P62/NRF2/HO-1 and the interaction of ROS with carcinogenesis will not only provide a better understanding of the pathophysiological mechanisms of tumorigenesis but can also lead to new ideas for tumor therapy and therapeutic targets.

## Materials and methods

### Cell culture and drug treatment

MCF-7 and MCF-7/ADR cell lines were obtained from Shanghai Zhongqiaoxinzhou Biotech and Jiangsu KeyGEN BioTECH Corp., Ltd, respectively. MCF-7 and MCF-7/ADR cells were cultured in MEM and RPMI-1640 medium supplemented with 10% fetal bovine serum (FBS) at 37 °C in a humidified incubator with 95% air and 5% CO_2_, respectively. Cells in the logarithmic growth phase were selected for subsequent experiments. 100 mM and 1 mg/mL stock solutions of Cyperotundone (CYT) (≥ 98%; Chengdu Alfa Biotechnology Co.Ltd. (Chengdu, China)) and Adriamycin (ADR) (98.0–102.0%; Zhejiang Hisun Pharmaceutical Co., Ltd) were prepared in dimethylsulfoxide (DMSO), respectively, and then diluted to different concentrations as indicated.

### Cell Viability Analysis

Cell Counting Kit-8 (CCK-8) assay was performed to detect cell viability. MCF-7 and MCF-7/ADR cells were seeded at 1 × 10^4^ cells/well and treated with 0.1, 0.2, 0.3, 0.4 , 0.5, or 0.6 mM CYT and with 0.078, 0.156, 0.313, 0.625, 1, 25, 2.5, 5, 10, 20, or 40 µg/mL ADR for 24 h, 48 h, or 72 h , respectively. The cells were incubated with CCK-8 at room temperature for 30 min-4 h. Subsequently, the optical density at 480 nm wavelength was measured with a multifunctional microplate reader (SpectraMax i3, Molecular Devices, San Jose, CA, USA), and the values were expressed as absorbance.

### Clone formation assay

The cells were inoculated onto a 6-well culture plate with a density of 500 cells/well, washed with PBS, and ADR, CYT, and ADR + CYT were added respectively according to the groups. The culture was terminated when clones were visible to the naked eye on the culture plate. The supernatant was removed and cells were washed with PBS twice, and then fixed with 1 mL 4% paraformaldehyde for 15 min. The fixing solution was then removed, and a sufficient amount of crystal violet staining solution was added and incubated for 15 min. The cells were then washed gently with running water and dried in the oven for several hours, and the image was taken using a WB strip instrument. Clone counting and result analysis: the number of clones was counted using the naked eye or ImageJ, and the clone formation rate was calculated as follows: Clone formation rate = number of clones / number of inoculated cells × 100%.

### Apoptosis analysis

Apoptotic cells were detected using Annexin V-FITC/Propidium Iodide (PI) staining according to the manufacturer’s protocol (BD Biosciences, San Diego, United States). 1 × 10^6^ MCF-7 and MCF-7/ADR cells were inoculated into a 6-well culture plate, respectively. The culture was terminated 24 h after treatment, the supernatant was discarded, and washed with PBS twice. Trypsin solution was added to detach the cells, followed by adding a double culture medium to stop digestion. The cells were then transferred to a centrifuge tube, centrifuged for 1500 r/5 min, and washed with PBS for 1500 r/5 min, twice. 100 μL 1× Buffer, 5 μL Annexin V-FITC, and 5 μL PI were added and incubated in the dark for 15–20 min. transferred the cells to the flow tube and added 300 μL 1× Buffer, apoptosis was detected by a flow cytometer (Becton Dickinson FACSCalibur, USA), and the data were analyzed by flowjo software.

### Cycle arrest analysis

1 × 10^6^ MCF-7 and MCF-7/ADR cells were inoculated onto a six-well culture plate, respectively. the culture was terminated at a specific time after treatment, the supernatant was discarded, and washed the cells were with PBS twice. Trypsin solution was added to detach the cells, followed by adding a double culture medium to stop digestion. the cells were then transferred to a centrifuge tube, centrifuged for 1000 r/3 min, washed with ice-cold PBS, and again centrifuged for 1000 r/3 min. After that, pre-cooled ethanol was added and fixed in a 4 °C refrigerator for ≥ 72 h. Then, centrifuge for 1000 r/3 min, and the supernatant was removed. The cells were washed once with PBS at 1000 r/3 min and 300 μL PI staining solution was added and incubated in the dark for 1 h before being filtered through filter paper into the flow tube. A flow cytometer (Becton Dickinson FACSCalibur, USA) was used to analyze the cell cycle. The data were analyzed by ModFit software.

### Production of reactive oxygen species (ROS)

Dichlorofluorescein diacetate (DCFH-DA) assay was performed to measure ROS generation. DCFH-DA can readily enter cells and be cleaved by esterase to yield DCFH, a nonfluorescent polar product. ROS in cells promotes the oxidation of DCFH and yields the fluorescent product dichlorofluorescein. MCF-7 and MCF-7/ADR cells were incubated in a six-well plate, respectively and the culture was terminated at a specific time after treatment. After the pretreatment period, cells were incubated with DCFH-DA at 37 °C in the culture medium for 30 min. The cells were then washed with PBS thrice. 200 μL of trypsin solution was added to each well to digest, the cells were then resuspended in 500 μL PBS and immediately analyzed through a flow cytometer (Becton Dickinson FACSCalibur, USA).

### Western blotting analysis

The total proteins were extracted with radioimmunoprecipitation assay (RIPA) lysis buffer. Equal concentrations of proteins were loaded on and separated by sodium dodecyl sulfate–polyacrylamide gel electrophoresis (SDS-PAGE) and then transferred onto polyvinylidene difluoride (PVDF) membranes. The membranes were incubated with primary antibodies and the corresponding secondary antibodies. Before incubating the primary antibody, cut the membrane according to the protein molecular weight.The primary antibodies including Bcl-2, Bax, Cleaved Caspase-3, cyclin D1, P16, P62, NRF2, HO-1, β-actin, and GAPDH were purchased from Abcam (Cambridge, UK). Then, ECL was applied to the membrane's protein surface and allowed to react for 3 min. Film exposure: 10 s to 5 min (exposure time was adjusted according to different light intensity), developed for 2 min and fixing. Image J software was used to analyze the gray value of the strip and calculate the relative gray value of the target strip (relative gray value = gray value/gray value of the reference strip of the same sample).

### Mouse xenograft model

A subcutaneous tumor model of breast cancer was established using both MCF-7 and MCF-7/ADR cell lines. 5–6-week-old female athymic balb/c nude mice were provided by Beijing Vital River Laboratory Animal Technology Co., Ltd. The animals were housed in a specific pathogen-free (SPF) environment and received food and water adlibitum. The SPF room is under a temperature of 22 ± 2 °C with a 12 h light/12 h dark cycle and relative humidity of 40–60%. After the mice had been in quarantine for 1 week, either 1 × 10^8^ MCF-7 cells suspended in a 100 μL mixture of PBS and matrigel (PBS: matrigel, 1:4) or 1 × 10^8^ MCF-7/ADR cells suspended in 100 μL PBS and matrigel were injected subcutaneously into the right flank of each mouse. When the tumor volume reached about 100 mm^3^, the mice were randomly divided into four groups and given intraperitoneal injections of normal saline, ADR, CYT (100 mg/kg/day), or ADR + CYT. The ADR dose of the MCF-7 group was 1 mg/kg/day, and that of the MCF-7/ADR group was 8 mg/kg/day, consistent with CCK-8. The weight of the mice and the volume of the tumors were measured. The mice were euthanized at the end of the experiment, and tumors were collected, photographed, and weighed.All animal experiment protocols were carried out following established ethical standards and with the approval of the Shandong Cancer Hospital and Institute (SDTHEC202100310Z). The experiment was completed before the tumor developed to the permissible maximum length/width of no more than 20 mm, as per guidelines. Tumor volume was calculated by a vernier caliper using the following formula: tumor volume length × width^2^/2.

### Immunohistochemical staining

The paraffin sections of mouse specimens were de-waxed and hydrated. Antigen restoration was processed for 5 min in a microwave oven with phosphate buffer. After applying the primary antibody, each slice was kept at 4 °C overnight. The secondary antibody was applied the next day and incubated at room temperature, stained with DAB for 5 min, and then counterstained with hematoxylin. Phosphate-buffered saline (PBS) was used as the negative control. The results were diagnosed and determined by two pathologists independently.

### Immunofluorescence assay

For immunofluorescence staining, paraffin-embedded tissue specimens of xenograft tumors were fixed with cold acetone and then permeabilized by 0.1% Triton X-100 in PBS. The tissue sections were incubated with primary antibodies at 4 °C overnight, followed by incubation with fluorescently labeled secondary antibodies. Nuclei were counterstained with DAPI. Images were taken with a fluorescence microscope and quantitated with ImageJ software.

### Statistical analysis

All datas were presented as mean ± S.D. and the statistical difference between groups was assessed using GraphPad Prism 9 software (GraphPad Software, Inc., San Diego, CA, United States). Paired Student’s *t*-test was used for the comparison of parameters between two groups. A *p-*value of < 0.05 was considered significant; For all tests, three levels of difference significance (*p < 0.05, ** *p* < 0.01) were applied.

### Ethics approval and consent to participate

Animal experiments were conducted in accordance with guide-lines of the Institutional Animal Care and Use Committee of the Shandong Institute of Materia Medica, Chinese Academy of Sciences. All animal studies were approved by the Committee on the Ethics of Animal Experiments of the Shandong Cancer Hospital (Permit Number: SDTHEC202100310Z). The study was conducted in accordance with the principles expressed in the Declaration of Helsinki. The study was carried out in compliance with the ARRIVE guidelines.

## Results

### Effects of CYT and ADR on cytotoxicity and proliferation of MCF-7 and MCF-7/ ADR cells

**Figure 1:**
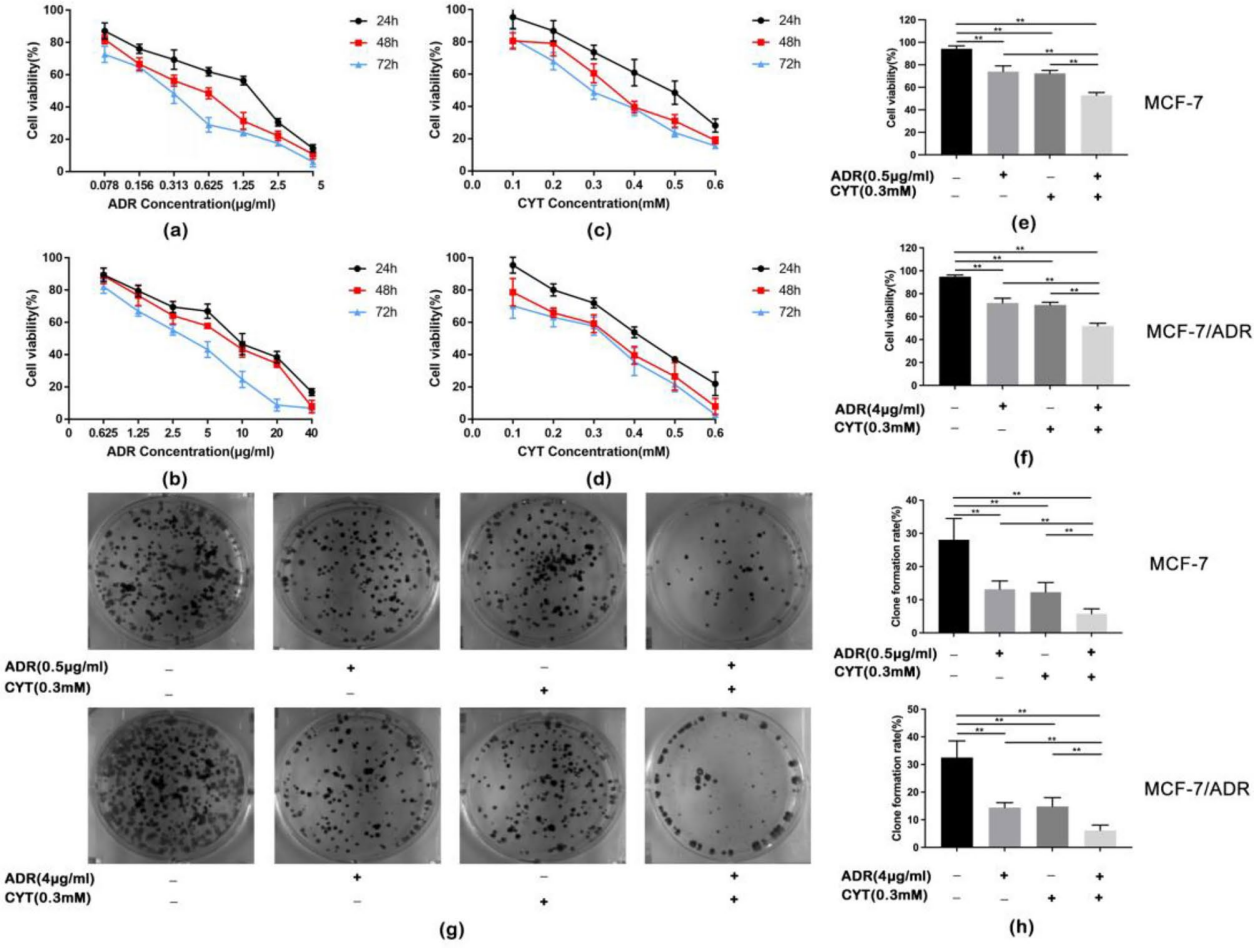
Effects of CYT and ADR on cytotoxicity and proliferation of MCF-7 and MCF-7/ADR cells. (**a,b**) CCK-8 assay showed that ADR inhibited the proliferation of MCF-7 and MCF-7/ADR cells (n = 6). (**c,d**) CCK-8 assay showed that CYT inhibited the proliferation of MCF-7 and MCF-7/ADR cells (n = 6). (**e,f**) ADR combined with CYT inhibited the proliferation of MCF-7 and MCF-7/ADR cells, as compared with the monotherapy group. The values are obtained from three independent experiments and shown as mean ± S.D. **p < .01. (**g,h**) The clone formation rates of the ADR, CYT, and ADR + CYT groups were lower than that of the control group, and the ADR + CYT group had the lowest rate in MCF-7 and MCF-7/ADR cells. The values are obtained from three independent experiments and shown as mean ± S.D. **p < .01.

CCK-8 assays revealed that CYT and ADR inhibited the viability of MCF-7 and MCF-7/ADR cells in a concentration and time-dependent manner, respectively (Fig. 1a–d). The calculated IC_50_ of MCF-7 and MCF-7/ADR at different time points are shown in Table 1a–d. The ADR effectiveness on MCF-7/ADR cells was about eight times that of MCF-7 cells, which was consistent with the instructions by the company. The higher the concentration of CYT and ADR, the lower the cell activity was observed. The concentration was selected based on the cell activity curves of ADR and CYT, where the cell activity was more than 70% and greater than the IC50 value of a single drug. For subsequent experiments, ADR 0.5 μg/ml + CYT 0.3 mM for MCF-7 cells and ADR 4 μg/ml + CYT 0.3 mM for MCF-7/ADR cells were used. Figure 1e,f demonstrated that the combination drug group inhibited breast cancer cell growth more effectively compared to the monotherapy group.

**Table 1:** The IC50 of MCF-7 and MCF-7/ADR at different times (mean ± SE).

(a)		(b)	
Time (h)	ADR IC50 (μg/ml)	Time (h)	ADR IC50 (μg/ml)
24	1.01 ± 0.03	24	8.76 ± 0.02
48	0.47 ± 0.02	48	6.36 ± 0.03
72	0.28 ± 0.02	72	3.04 ± 0.02

(c)		(d)	
Time (h)	CYT IC50 (mM)	Time (h)	CYT IC50 (mM)
24	0.40 ± 0.01	24	0.46 ± 0.01
48	0.29 ± 0.02	48	0.34 ± 0.01
72	0.26 ± 0.03	72	0.28 ± 0.01

(a) IC_50_ of ADR against the MCF-7 cells at 24, 48, and 72 h; (b) IC_50_ of ADR against the MCF-7/ADR cells at 24, 48, and 72 h; (c) IC_50_ of CYT against the MCF-7cells at 24, 48 and 72 h; (d) IC_50_ of CYT against the MCF-7/ADR cells at 24, 48, and 72 h.

Cell proliferation was determined by clone formation assay. For MCF-7, the clone formation rates of the ADR and CYT groups were significantly lower than that of the control group (DMSO) (*p* < 0.01). Clone formation rates of the ADR, CYT and ADR + CYT groups were lower than that of the control group, and the ADR + CYT group showed the lowest rate of clone formation (*p* < 0.01). The clone formation rates in the other three groups were lower after drug treatment in MCF-7/ADR than in the control group. The clone formation rate of the combined therapy was significantly lower than those of the ADR and CYT monotherapy groups (*p* < 0.01). Collectively, CYT and ADR inhibited the proliferation of MCF-7 and MCF-7/ADR cells after appropriate treatment time at certain concentrations, while ADR + CYT inhibited the proliferative potential even more (Fig. 1g,h).

### CYT combined with ADR intensified apoptosis and inhibited the cell cycle in the G0 / G1 phase of MCF-7 and MCF-7/ADR cells

**Figure 2:**
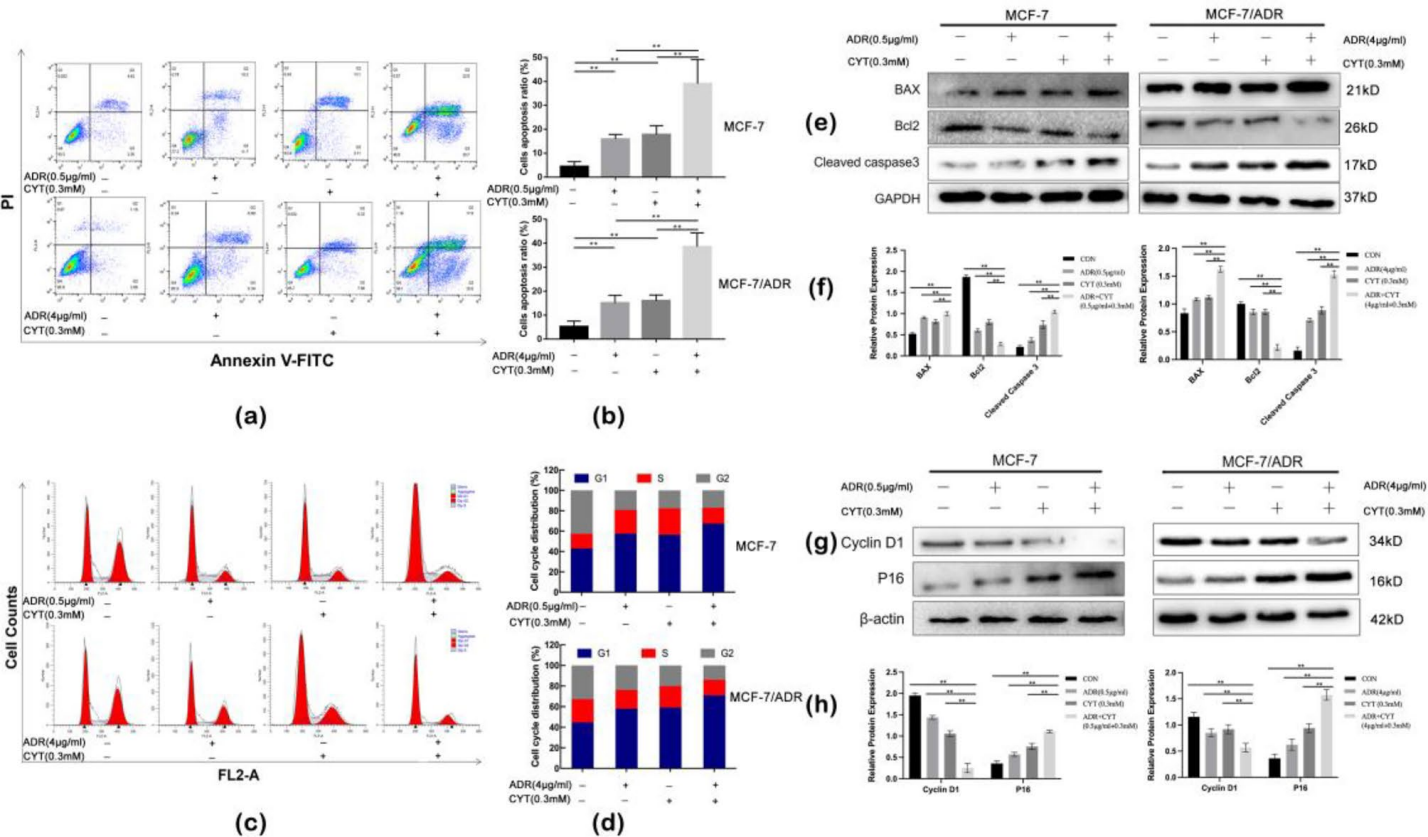
CYT combined with ADR intensified apoptosis and inhibited the cell cycle in the G0/G1 phase of MCF-7 and MCF-7/ADR cells. (**a,b**) Flow cytometry indicated that the apoptosis rate of the monotherapy group was higher than that of the control group in MCF-7 and MCF-7/ADR cells, the apoptosis rate of the ADR + CYT group was significantly higher than that of the monotherapy group. (**c,d**) Flow cytometry indicated that G0/G1 phases in the monotherapy group and the combined group were greater than that in the control group in MCF-7 and MCF-7/ADR cells. (**e**) The antiapoptotic protein Bcl-2 was lower in the combined group, but proapoptotic proteins, such as BAX and Cleaved-caspase3, were significantly increased. (**f**) The relative expression of the apoptotic proteins (Bcl-2/GAPDH, BAX/GAPDH, and Cleaved-caspase3/GAPDH) were quantified by ImageJ and analyzed by GraphPad Prism 9. (**g**) Western blot analysis to determine the expression of P16, Cyclin D1. β-actin was used as a loading control. (**h**) The relative expression of G0–G1 phase transition-related proteins Cyclin D1/β-actin and P16/β-actin) was quantified by ImageJ and analyzed by GraphPad Prism 9. The values are obtained from three independent experiments and represented as mean ± S.D. **p < .01.

In MCF-7 and MCF-7/ADR cells, early apoptosis of quadrant 2 and late apoptosis of quadrant 3 in the CYT and ADR combined groups were significantly higher than those in the control group. Similarly, both results were higher than for monotherapy group, and the differences were statistically significant (Fig. 2a,b) (*p* < 0.01). Furthermore, cell cycle analysis was performed through flow cytometry to see whether CYT combined with ADR may cause cell cycle arrest. As shown in Fig. 2c,d, after ADR, CYT, and ADR + CYT treatment for 24 h, ADR + CYT further inhibited the G0/G1 phase of breast cancer cells.

The expression of apoptosis-related proteins was detected by western blot. The antiapoptotic protein Bcl-2 was decreased in the ADR + CYT group in MCF-7 and MCF-7/ADR cells than in the control and monotherapy groups. Whereas, the proapoptotic proteins BAX and cleaved-caspase3 were increased (Fig. 2e,f). Original blots/gels are presented in Supplementary Figs. 1–8.The combination of CYT and ADR promoted apoptosis when compared with the monotherapy group, which was consistent with the findings of flow cytometry experiments. Western blots were used to detect the expression of cyclin-related proteins. The findings revealed that the combination therapy considerably decreased cyclin D1 expression while increasing P16 expression in MCF-7 and MCF-7/ADR cells (Fig. 2g,h). Original blots/gels are presented in Supplementary Figs. 9–14.Compared with the monotherapy groups, the combination of CYT and ADR triggered G0/G1 phase arrest, which was consistent with flow cytometry findings.

### CYT combined with ADR increases the production of ROS in MCF-7 and MCF-7/ADR cells

**Figure 3:**
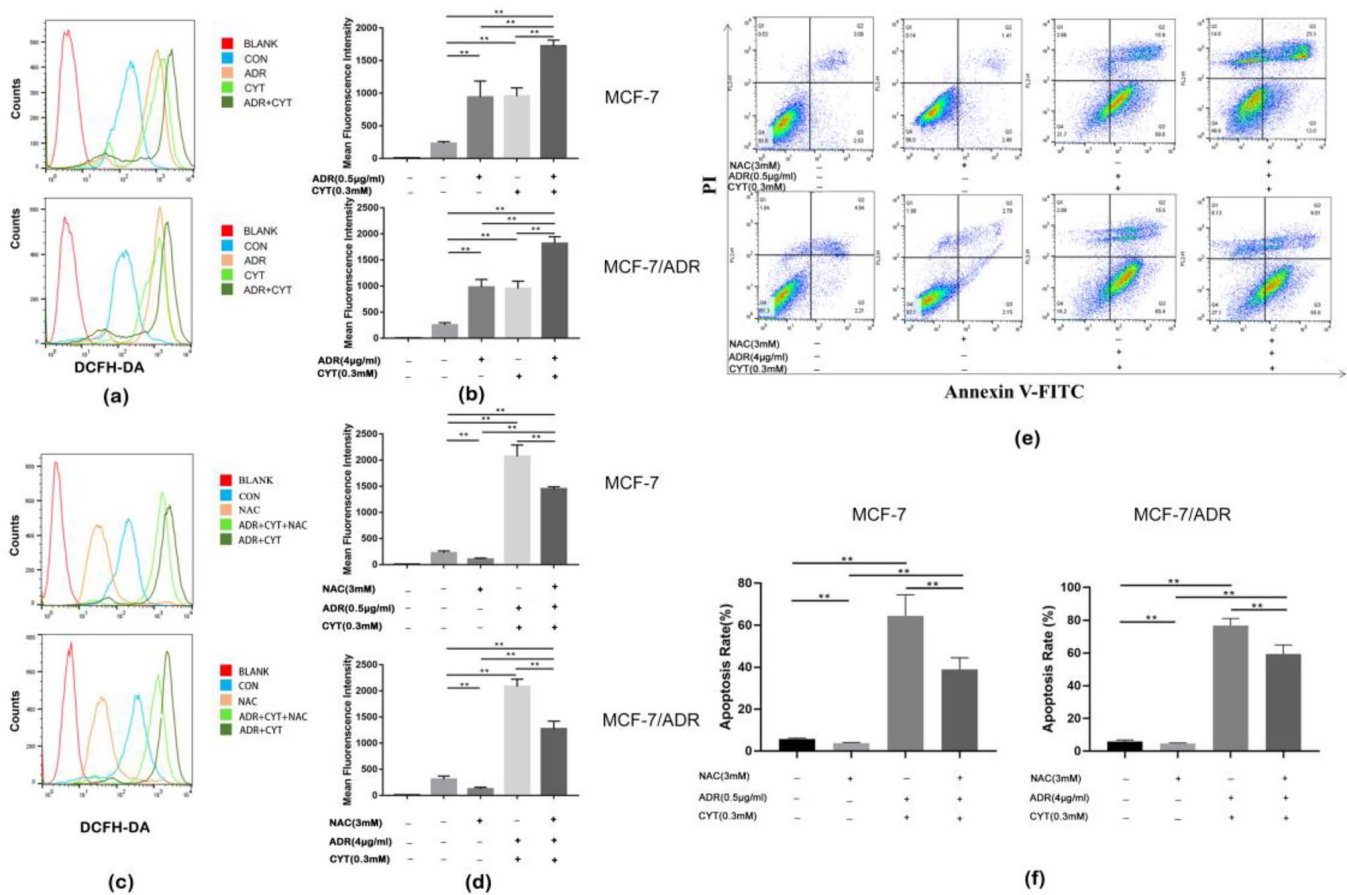
CYT combined with ADR increases the production of ROS in MCF-7 and MCF-7/ADR cells. (**a**) Flow cytometry results of ADR + CYT induced ROS generation in MCF-7 and MCF-7/ADR cells. (**b**) The bar chart showed the mean ± S.D. of three experiments. **p < .01 vs control. (**c**) NAC inhibited ADR + CYT induced ROS generation in MCF-7 and MCF-7/ADR cells. (**d**) The bar chart showed the mean ± S.D. of three experiments. (**e**) NAC inhibited ADR + CYT induced apoptosis in MCF-7 and MCF-7/ADR cells. (**f**) The bar chart showed the mean ± S.D. of three experiments. **p < .01.

Since the proapoptotic effect has traditionally been associated with oxidative stress^21^, we studied ROS production in MCF-7 and MCF-7/ADR cells to validate the oxidative stress induced by CYT combined with ADR. As shown in (Fig. 3a,b), CYT combined with ADR significantly increased the production of intracellular ROS in MCF-7 and MCF-7/ADR cells after 0.5 h incubation with DCFH-DA. NAC (*N*-acetyl-l-cysteine), a ROS inhibitor, significantly reduced the CYT combined with ADR-induced ROS generation in MCF-7 cells and MCF-7/ADR cells (Fig. 3c,d). These results suggested that CYT combined with ADR could induce oxidative injury in MCF-7 cells and MCF-7/ADR cells, whereas NAC could significantly reverse CYT combined with ADR-induced ROS generation. We used flow cytometry to detect the apoptosis rate of each group after adding NAC to further validate the relationship between ROS and apoptosis. The findings revealed that NAC substantially reduced the rate of apoptosis in the NAC group, suggesting that ROS generation might cause apoptosis and enhance chemotherapy sensitivity (Figs. 3e, 5f).

### CYT combined with ADR Induced MCF-7 and MCF-7/ADR cells apoptosis by inhibiting the NRF2/ARE signaling pathways

**Figure 4:**
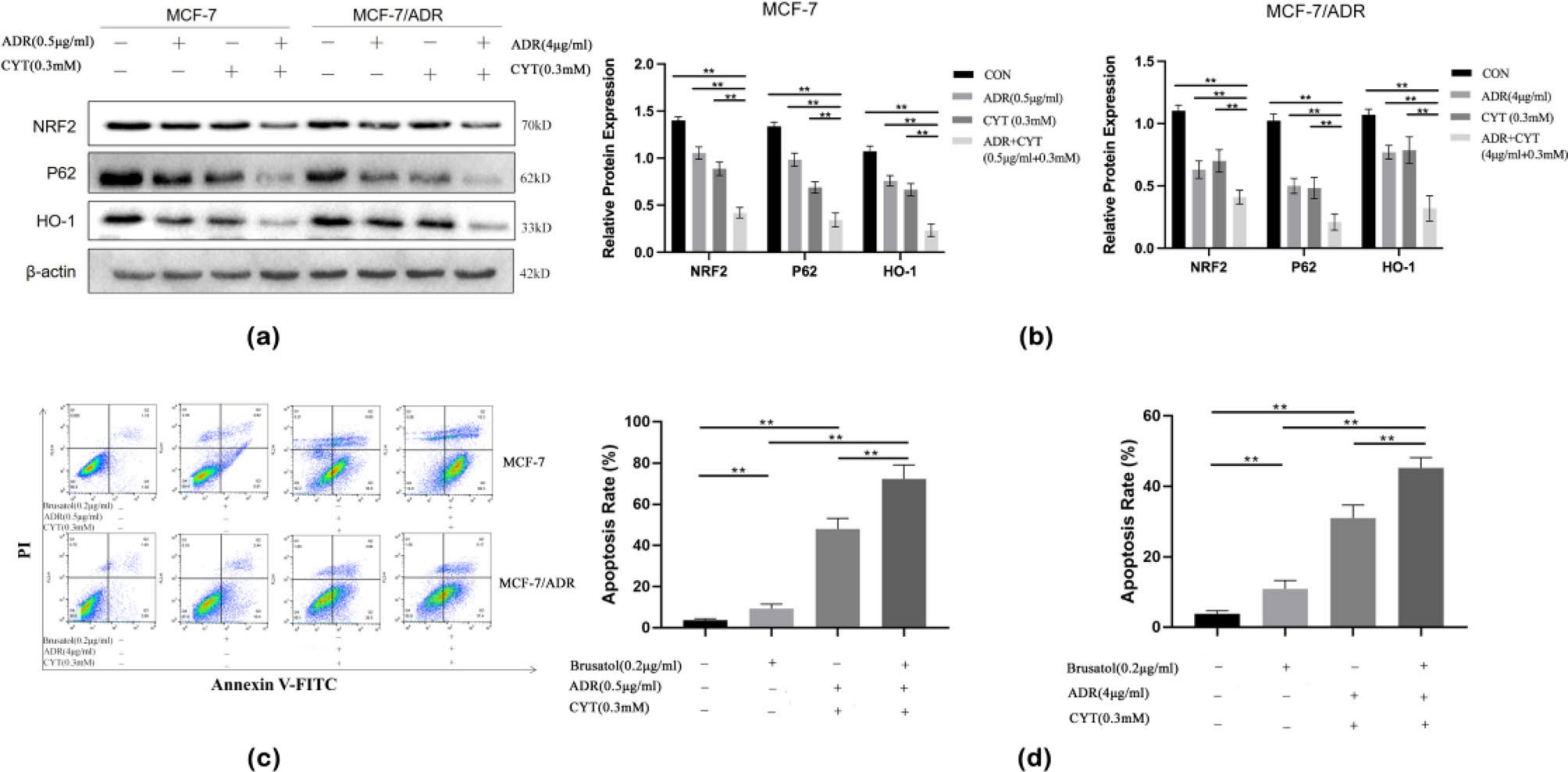
CYT combined with ADR Induced MCF-7 and MCF-7/ADR cells apoptosis by inhibiting the NRF2/ARE signaling pathways. (**a**) ADR + CYT reduced the expression levels of P62, NRF2, and HO-1 in MCF-7 cells and MCF-7/ADR cells. (**b**) The bar chart showed the mean ± S.D. of three experiments. β-actin was the internal control. (**c**) Brusatal promotes ADR + CYT-induced apoptosis in MCF-7 and MCF-7/ADR cells. (**d**) The bar chart showed the mean ± S.D. of three experiments.**p < .01.

The CYT and ADR groups exhibited lower P62 levels than the control group in both MCF-7 and MCF-7/ADR cells, and the combination treatment had significantly lower P62 levels than the single-drug treatment (P < 0.01). NRF2 expression was downregulated in the CYT and ADR groups compared to the control group (*p* < 0.05) and was significantly downregulated in the combination group compared to the single drug group (*p* < 0.01). The expression of HO-1 in the combined group was also significantly lower than in the CYT and ADR groups (*p* < 0.01) (Fig. 4a). Original blots/gels are presented in Supplementary Fig. 15–18.The expression level of related proteins are shown in Fig. 4b. We employed the NRF2 inhibitor brusatal to evaluate the effect of CYT combined with ADR on apoptosis to further verify the involvement of NRF2 in CYT combined with ADR. The findings revealed that brusatal significantly increased ADR + CYT induced apoptosis (Fig. 4c, d), suggesting the involvement of NRF2 in cancer cell apoptosis.

### CYT combined with ADR exhibited significantly inhibitory effects in tumor-bearing nude mice

**Figure 5:**
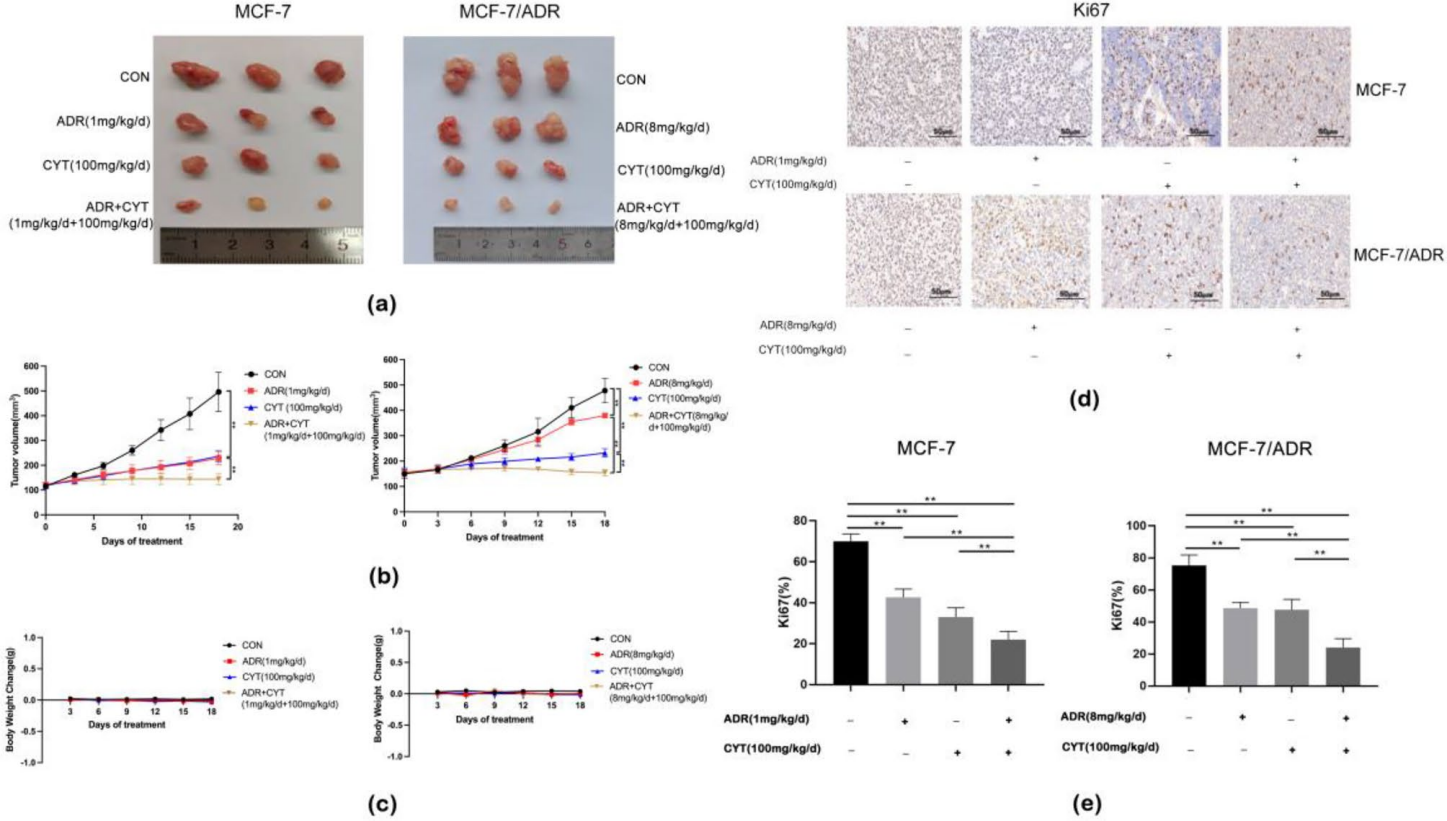
Anti-tumor effects of ADR + CYT on MCF-7 and MCF-7/ADR xenograft tumor model. (**a**) The photographs of tumors of MCF-7 and MCF-7/ADR after treatment with saline, ADR, CYT, and ADR + CYT. (**b**) Tumor volume was measured in different time intervals using a vernier caliper and calculated. (**c**) Body weight change of all groups. (**d**) The expression of Ki67 was detected by immunohistochemistry. (**e**) Data were expressed as mean ± S.D. **p < .01.

In *vivo* experiments revealed that tumor volume growth in the CYT and ADR groups was marginally slower than that in the control group (*p* < 0.01). While the combination treatment group showed a considerably slower tumor volume growth than that of the control, CYT, and ADR groups, and tumor volume was significantly decreased (Fig. 5a,b) (*p* < 0.01). In all groups, there was no significant change found in body weight (Fig. 5c). Furthermore, we also examined the hepatorenal toxicity of CYT. According to our findings, the hepatorenal toxicity indices of the CYT treatment group and the control group were both within the normal range (Table 2).

**Table 2:** Liver and kidney toxicity indexes of CYT treatment group and control group.

	ALT	AST	BUN	CR	UA
CON	47.67 ± 8.40	111.69 ± 13.23	11.34 ± 0.38	11.95 ± 0.76	92.33 ± 1.98
CYT	67.57 ± 12.09	173.36 ± 15.53	17.99 ± 0.81	15.84 ± 0.55	175.76 ± 12.24

The normal reference range of each index: alanine aminotransferase (ALT) (10.06–96.4U/L), aspartate aminotransferase (AST) (36.31–235.48U/L), blood urea nitrogen (BUN) (10.81–34.70 mg/dL), creatinine (CR) (10.91–85.09 μmol/L), Uric acid (UA) (44.42–224 μmol/L). Control group (CON) vs.cyperotundone group (CYT).

Immunohistochemistry demonstrated that the expression of Ki-67 was lower in the CYT group and ADR group than in the control group, and significantly lower in the combination group (*p* < 0.01) (Fig. 5d,e). The results indicated that tumor proliferation was significantly decreased after combined CYT and ADR therapy.

### CYT combined with ADR exhibited significantly inhibitory effects in tumor-bearing nude mice by ROS generation

**Figure 6:**
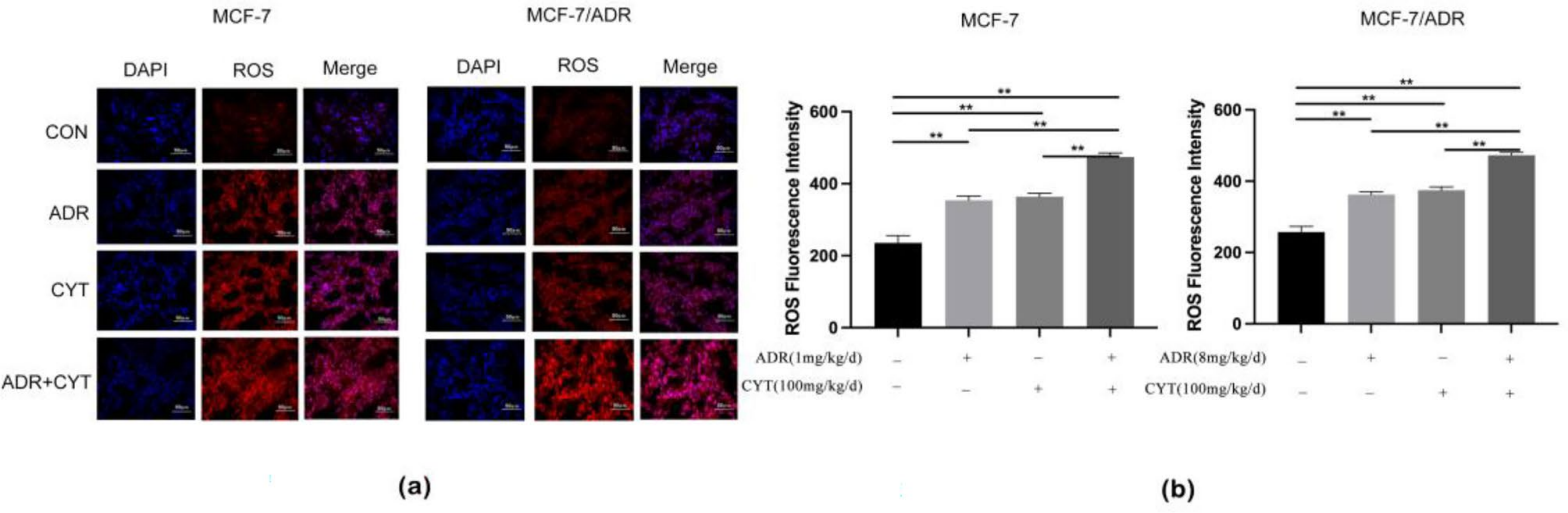
CYT combined with ADR exhibited significantly inhibitory effects in tumor-bearing nude mice by ROS generation. (**a**) The expression of ROS in tissues was detected by immunofluorescence. (**b**) The fluorescence intensity of ROS was detected by image J.

We used immunofluorescence to detect the ROS generation in nude mouse tumor tissues to further investigate the mechanism of ADR combined with CYT in inhibiting tumors in nude mice. The results showed that the expression of ROS in ADR combined with the CYT group was significantly higher than that in the control group and single-drug group (Fig. 6a,b).

## Discussion

According to statistics, breast cancer is the most commonly diagnosed cancer globally in 2020. Despite recent breakthroughs in diagnostic technologies and early diagnosis, recurrence rates for early breast cancer are ranged from 7 to 18%, according to various studies. Recurrent breast cancer is associated with a worse prognosis and higher mortality rates^22^. Drug resistance is a major cause of tumor recurrence, however, the underlying mechanism is still largely unclear^23^. Tumor heterogeneity and adaptability, which contribute to chemotherapeutic drug resistance, are still important concerns in cancer therapy^24,25^. Even though many established mechanisms behind antitumor drug resistance, none can completely explain multidrug-resistant cancer in patients with advanced cancer. Among the identified mechanisms of chemoresistance, the composition of the plasma membrane, overexpression of efflux pump, modification of drug metabolism by detoxification enzymes, increasing the ability of cells to repair DNA damage or tolerate stress conditions, and loss of function of proapoptotic factors have attracted the most attention so far^26–28^. Recently, oxidative stress has been found as a crucial component in chemoresistance control, although the mechanism still needs to be understood^9,10^. Adriamycin hydrochloride (ADR) is a common chemotherapeutic drug for the treatment of a variety of cancers. However, cardiotoxicity in clinical treatment is the main side effect of ADR. In addition, the emergence of multidrug resistance (MDR) in tumor cells reduces the efficiency of ADR^28^.

Anxiety, irritability, depression, and other emotional disorders are common among breast cancer patients^29,30^. Traditional Chinese Medicine such as soothing the liver, relieving stagnation, and activating blood circulation is usually used as an adjunct treatment for breast cancer and has a good effect on the survival rate and prognosis of patients^31–35^. However, the mechanism of this enhanced therapeutic effect has not been studied. In Chinese medicine, Rhizoma cyperi could promote the flow of Qi in the liver and Sanjiao channels, regulate menstruation, and alleviate pain. Clinically, Rhizoma cyperi is used for depression, flatulence, hypochondriac pain, and dysmenorrhea. Thus, it has a long history and significant curative effect on the treatment of various Qi stagnation symptoms^36^. Recent research has shown that Rhizoma Cyperi or Cyperus has anticancer effects; moreover, our preliminary studies have verified the antitumor effect of EECR on triple-negative breast cancer^37,38^. Cypermethenone is the main active ingredient of Cyperus, but its antitumor effect on breast cancer has not been reported. As a result, we hypothesized that cypermethene combined with adriamycin suppresses HR-positive breast cancer cells as well as drug-resistant cells. Our research confirmed the antitumor effect of ADR + CYT in breast cancer. ADR + CYT both significantly inhibited the malignant proliferation of MCF-7 and MCF-7/ADR cells in *vitro* and in *vivo*. In this study, it was found that ADR + CYT significantly induced apoptosis of MCF-7 and MCF-7/ADR in *vitro*, which was characterized by increased expression of activated caspase3, up-regulation of Bax, and downregulation of antiapoptotic Bcl2 expression.

Oxidative stress is defined as the excessive generation of ROS, RONs, and other free radicals in the body as a result of numerous damaging stimuli, which surpasses the body's scavenging capability and disrupt the equilibrium of oxidants/antioxidants^39^. When it comes to the normal body, oxidative stress may be harmful, but it may play an important role in tumor cells^40^. NRF2 is one of the important pathways for intracellular antioxidant stress and maintenance of redox. NRF2 also plays a "double-edged sword" role in tumor cells. On the one hand, the activation of the NRF2/ARE pathway is favorable to resisting bodily harm and inhibiting tumor formation when the body is stimulated by internal and external constraints under normal conditions. Overactivated NRF2, on the other hand, may enhance tumor growth, metastasis, and drug resistance^17–19^. NRF2 protein levels are consistently raised in different kinds of cancer cells and tumor specimens from cancer patients, and high expression of NRF2 in tumor tissues is associated with a poor prognosis^41,42^. NRF2 activation in cancer cells is closely related to multiple pathways, such as NRF2 gene mutation leading to overactivation of NRF2 in cancer cells; P62 accumulation competes with NRF2 to bind Keap1 to that NRF2 accumulates in tumor cells. In addition, the activation of pathways such as MAPK and PERK also activates the NRF2 pathway^43–45^. It is worth noting that the positive feedback loop formed by NRF2 and P62 may be the key pathway of carcinogenic factors.

Studies have shown that P62, NRF2, and HO-1 may interact with each other. For instance, The use of targeted siRNA to silence either NRF2 or P62/SQSTM1 revealed a crosstalk between the two molecules and that knocking down either molecule enhanced cancer cell sensitivity to Zn(II)–curc-induced cell death^46^. Furthermore, the combined treatment of luteolin and paclitaxel showed an enhanced cytotoxic effect on breast cancer stem cells via decreasing NRF2 expression^47^. Previous studies have also found that the expressions of NRF2 and p62 in breast cancer were higher than those in the corresponding adjacent normal tissues and benign breast epithelial cells. The expressions of NRF2 and P62 in breast cancer doxorubicin-resistant cells MCF-7/ADR were higher than those in doxorubicin-sensitive MCF-7 cells. Silencing of NRF2 or P62 rendered breast cancer cells more susceptible to doxorubicin^48^. The expression level of P62 in cisplatin-resistant human ovarian cancer cells is much higher than that in cisplatin-sensitive cells, and cisplatin resistance is acquired through regulating the keap1-NRF2-ARE signal pathway. P62 knockout in cisplatin-resistant human ovarian cancer cells can restore cisplatin sensitivity^49,50^. Our study showed that ADR + CYT significantly reduced the expression of P62, NRF2, and HO-1 proteins in MCF-7 and MCF-7/ADR breast cancer cells.

In this study, we found that ADR + CYT significantly inhibited the proliferation of MCF-7 and MCF-7/ADR breast cancer cells, promoted apoptosis, blocked the cell cycle at the G0/G1 stage, reduced the expression of P62, NRF2, and HO-1, and enhanced chemosensitivity. We initially hypothesized that ADR + CYT would improve chemosensitivity by modulating the P62/NRF2/HO-1 pathway. However, P62/NRF2/HO-1 pathway is complex and rigorous, and a more rigorous experimental design is required to thoroughly investigate its mechanism in cancer, to provide a more theoretical basis for preventing and delaying the development of tumors, and provide new ideas for finding tumor treatment and the target of new drugs.

## Conclusion

**Figure 7:**
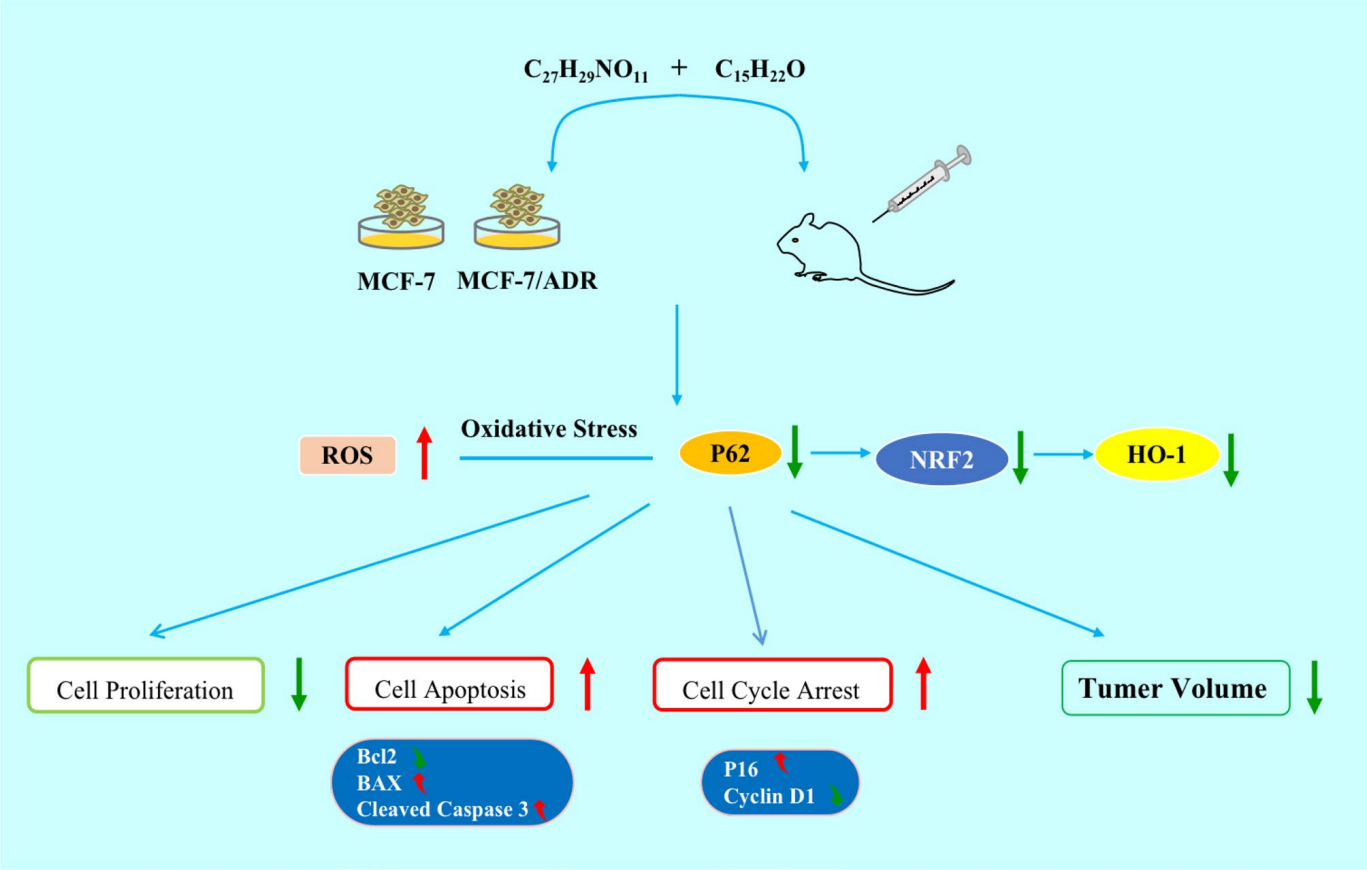
A schematic diagram of the effect of ADR + CYT combination on MCF-7 and MCF-7/ADR cells and their tumor bearing nude mice.

This research demonstrated that CYT in combination with ADR cytotoxic to MCF-7 and MCF-7/ADR cells(Fig. 7). The combination of CYT and ADR significantly inhibited MCF-7 cell growth by promoting cell cycle arrest and apoptosis. Increased ROS formation, regulation of Bcl-2 family proteins, and activation of caspase3 may all play a role in inducing apoptosis. The combination of CYT and ADR inhibits the activation of the P62/NRF2/HO-1 pathway, which contributes to the activation of the apoptotic pathway. Meanwhile, the antitumor effect of CYT in combination with ADR has been mostly verified in *vivo* on MCF-7 cells and MCF-7/ADR cells xenografts, with no noticeable toxicity at a dose of 100 mg/kg/day after 15 days of therapy. CYT in combination with ADR inhibited tumor development possibly through ROS generation. These findings suggest that CYT may be developed as a possible therapy to increase the sensitivity of breast cancer chemotherapy.

## Supplementary Information

Supplementary Figure 1.

Supplementary Figure 2.

Supplementary Figure 3.

Supplementary Figure 4.

Supplementary Figure 5.

Supplementary Figure 6.

Supplementary Figure 7.

Supplementary Figure 8.

Supplementary Figure 9.

Supplementary Figure 10.

Supplementary Figure 11.

Supplementary Figure 12.

Supplementary Figure 13.

Supplementary Figure 14.

Supplementary Figure 15.

Supplementary Figure 16.

Supplementary Figure 17.

Supplementary Figure 18.

## Data Availability

The research data used to support the findings of this study are included within the article.

## Abbreviations

CYT: Cyperotundone
ADR: Adriamycin
ROS: Reactive oxygen species
NRF2: Nuclear factor E2-related factor 2
HO-1: Heme oxygenase-1
CCK8: Cell counting kit 8
PI: Propionate iodide
IC50: Half maximal inhibitory concentration
SD: Standard deviation
TCM: Traditional Chinese medicine
MCF-7/ADR: MCF-7 adriamycin resistant cells
DMSO: Dimethyl sulfoxide
DCFH-DA: Dichlorofluorescein diacetate
RIPA: Radioimmunoprecipitation assay
PBS: Phosphate-buffered saline
NAC: *N*-Acetyl-l-cysteine
GAPDH: Glyceraldehyde 3-phosphate dehydrogenase
ANOVA: Analysis of variance
