# Supplementary figures and images for: Cyperotundone combined with adriamycin induces apoptosis in MCF-7 and MCF-7/ADR cancer cells by ROS generation and NRF2/ARE signaling pathway
Source: Sci Rep. 2023 Jan 25;13:1384. doi: 10.1038/s41598-022-26767-x (PMC9877033; doi:10.1038/s41598-022-26767-x)

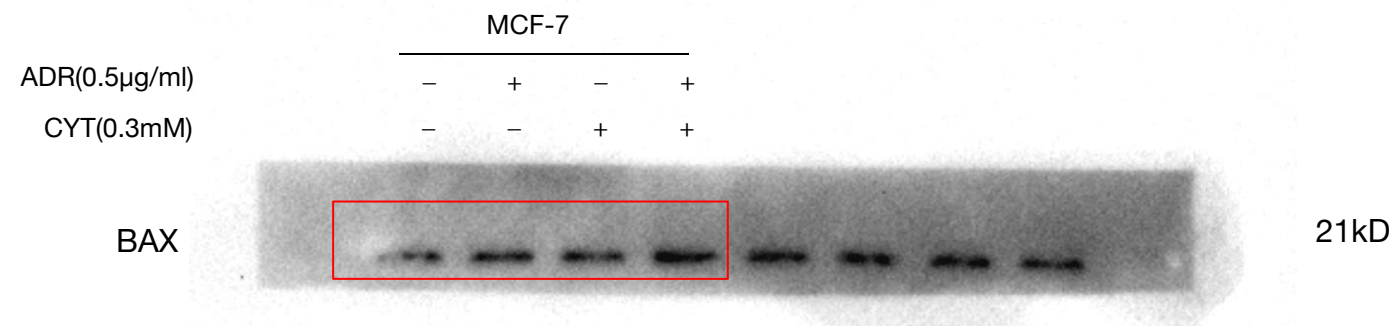

Supplementary Figure 1: Original for Figure 2(e).

Supplement: Supplementary file 1 — Supplementary Figure 1. [file 41598_2022_26767_MOESM1_ESM.pdf]

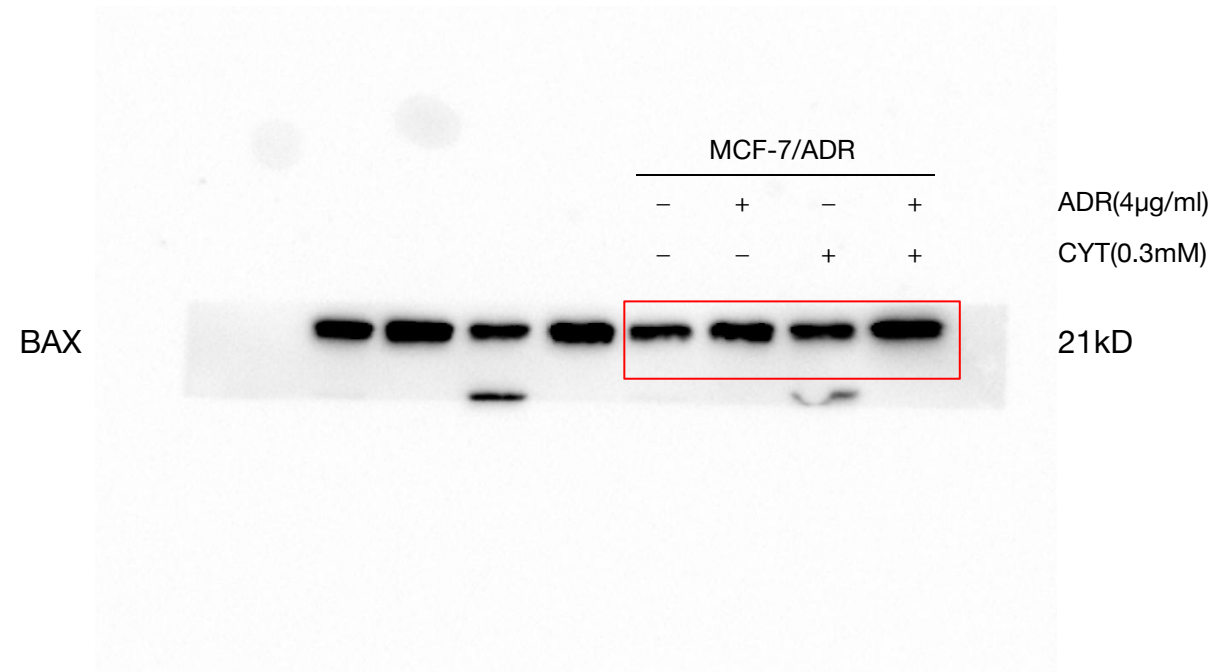

Supplementary Figure 2: Original for Figure 2(e).

Supplement: Supplementary file 2 — Supplementary Figure 2. [file 41598_2022_26767_MOESM2_ESM.pdf]

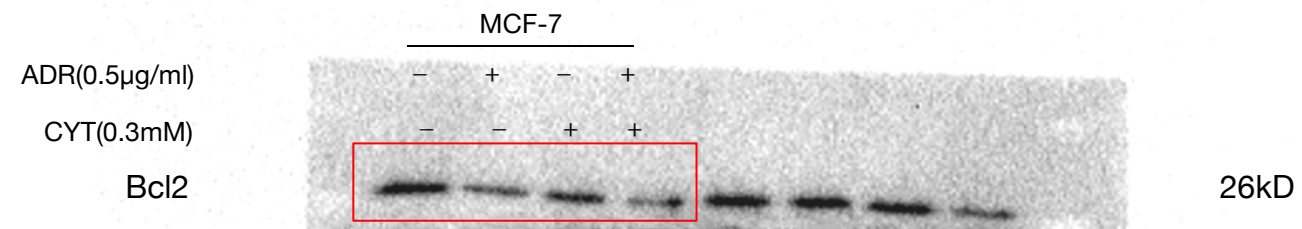

Supplementary Figure 3: Original for Figure 2(e).

Supplement: Supplementary file 3 — Supplementary Figure 3. [file 41598_2022_26767_MOESM3_ESM.pdf]

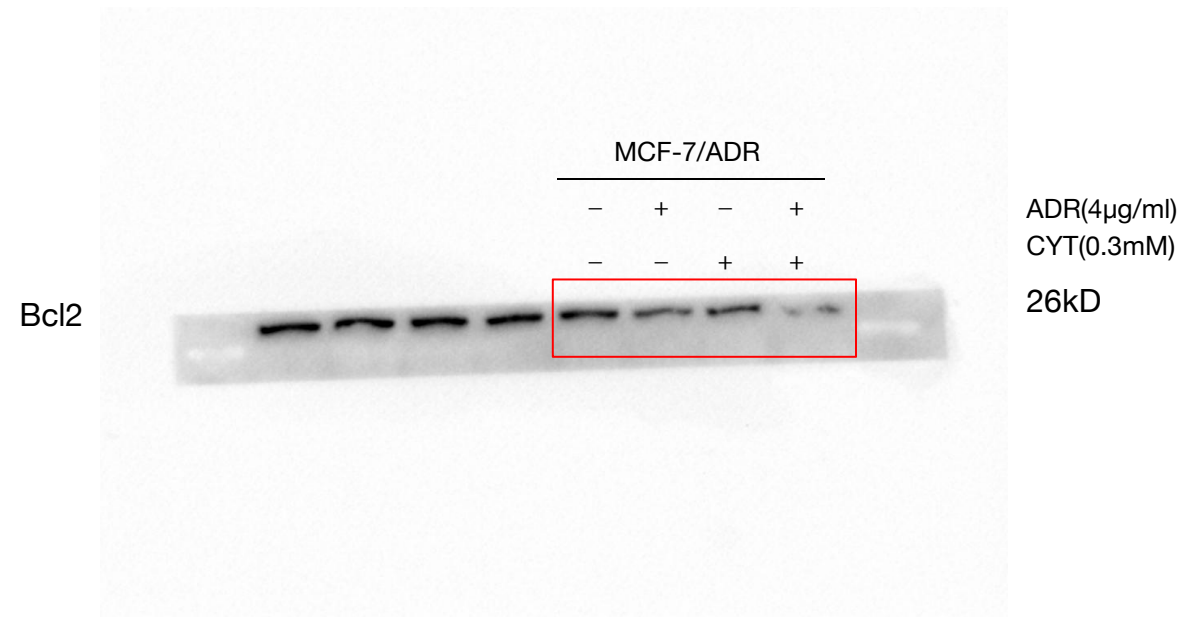

Supplementary Figure 4: Original for Figure 2(e).

Supplement: Supplementary file 4 — Supplementary Figure 4. [file 41598_2022_26767_MOESM4_ESM.pdf]

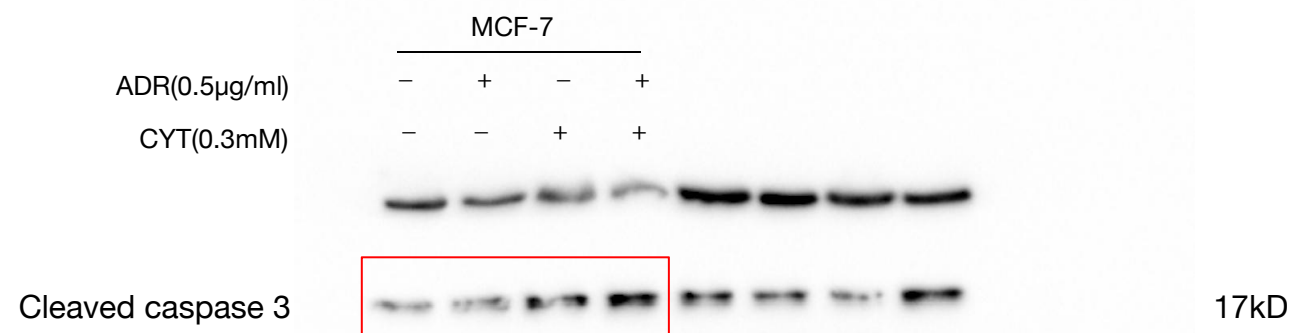

Supplementary Figure 5: Original for Figure 2(e).

Supplement: Supplementary file 5 — Supplementary Figure 5. [file 41598_2022_26767_MOESM5_ESM.pdf]

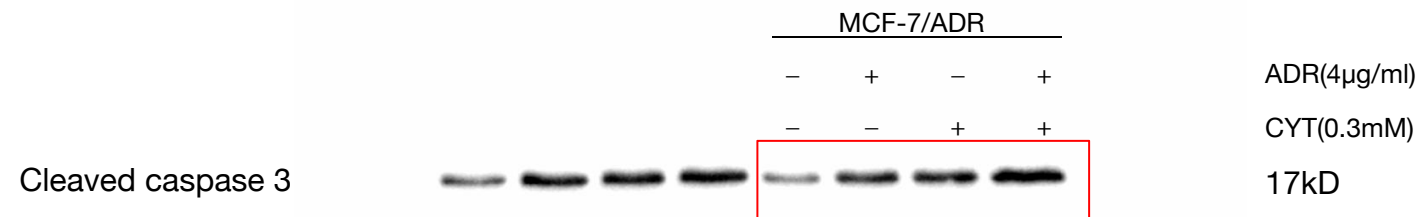

Supplementary Figure 6: Original for Figure 2(e).

Supplement: Supplementary file 6 — Supplementary Figure 6. [file 41598_2022_26767_MOESM6_ESM.pdf]

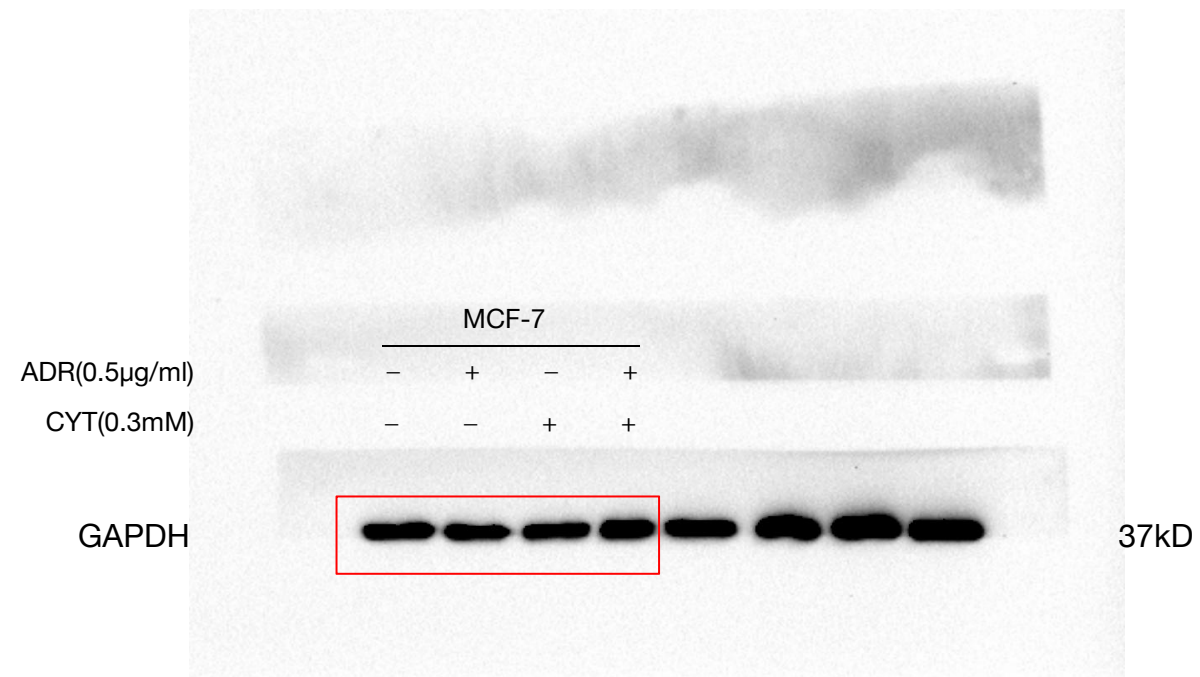

Supplementary Figure 7: Original for Figure 2(e).

Supplement: Supplementary file 7 — Supplementary Figure 7. [file 41598_2022_26767_MOESM7_ESM.pdf]

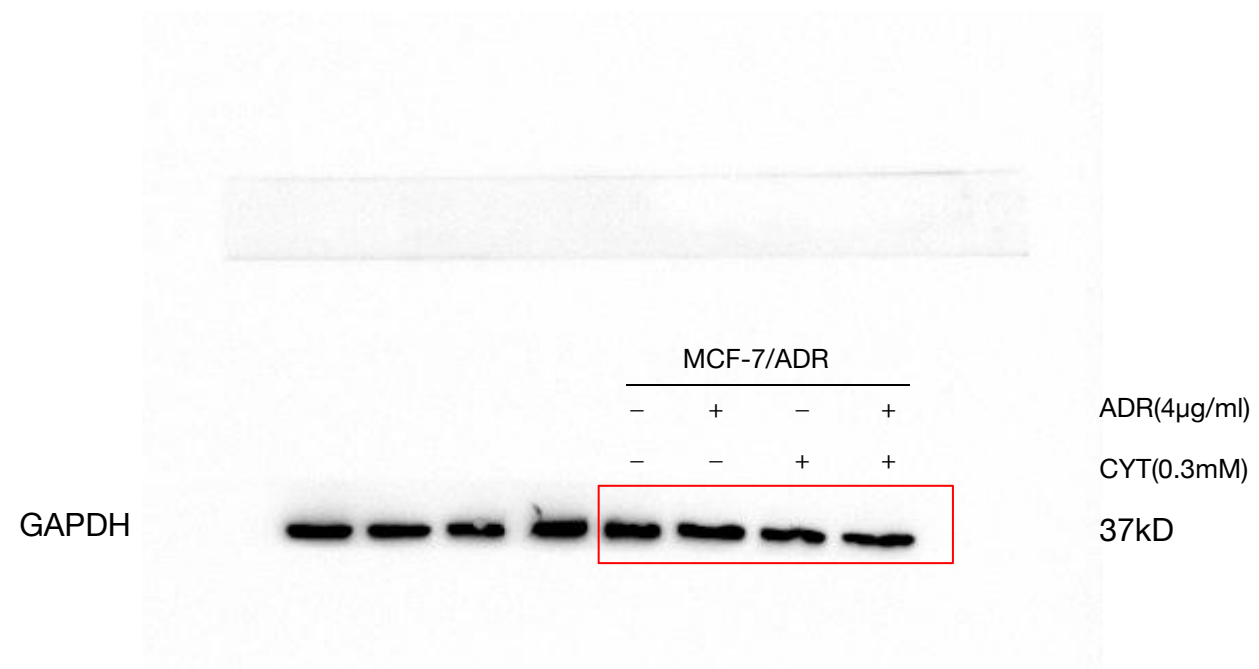

Supplementary Figure 8: Original for Figure 2(e).

Supplement: Supplementary file 8 — Supplementary Figure 8. [file 41598_2022_26767_MOESM8_ESM.pdf]

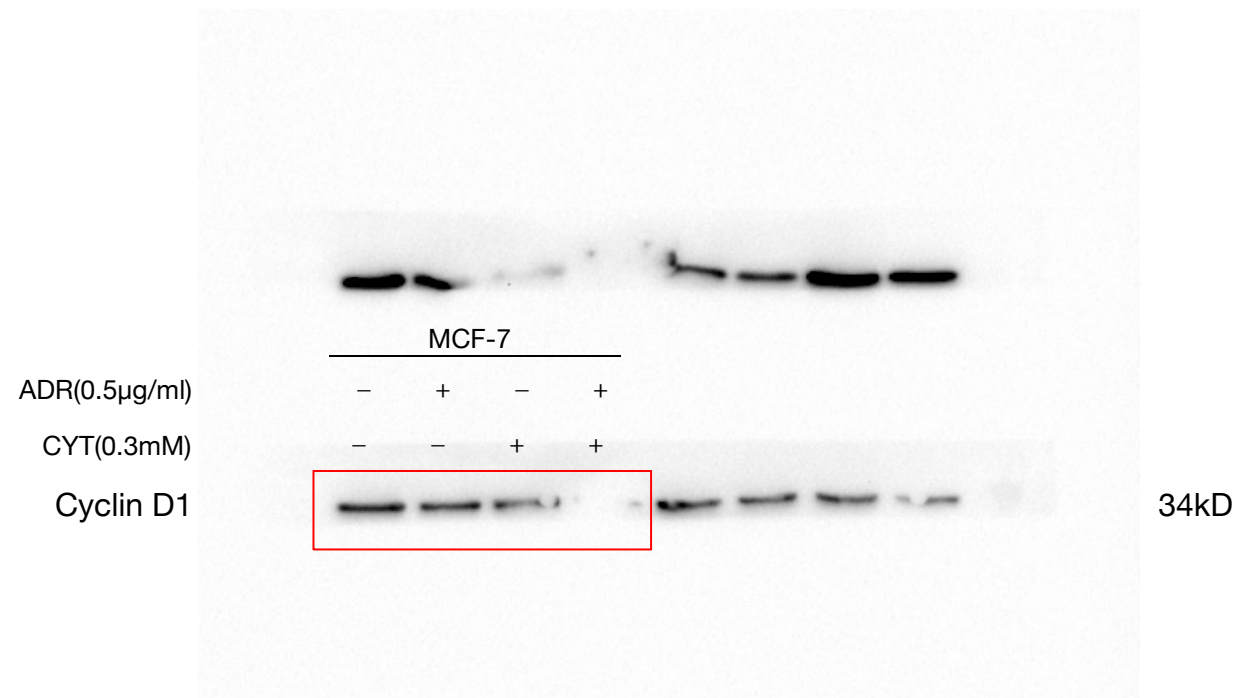

Supplementary Figure 9: Original for Figure 2(g).

Supplement: Supplementary file 9 — Supplementary Figure 9. [file 41598_2022_26767_MOESM9_ESM.pdf]

Cyclin D1

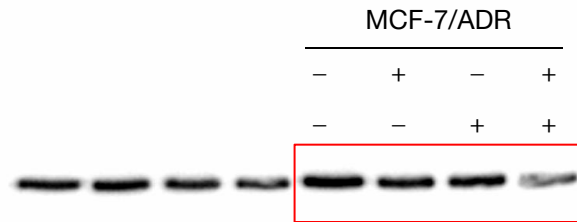

ADR(4 $\mu$ g/ml)

CYT(0.3mM)

34kD

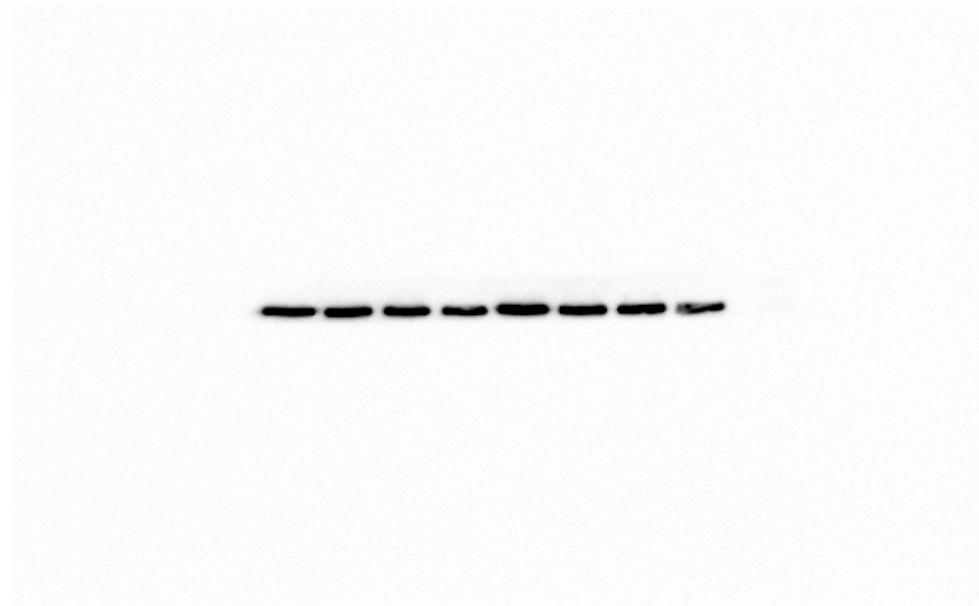

Supplementary Figure 10: Original for Figure 2(g).

Supplement: Supplementary file 10 — Supplementary Figure 10. [file 41598_2022_26767_MOESM10_ESM.pdf]

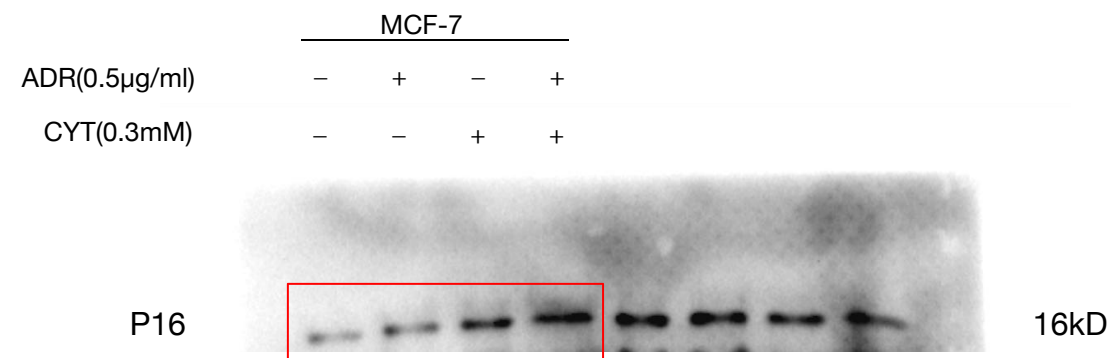

Supplementary Figure 11: Original for Figure 2(g).

Supplement: Supplementary file 11 — Supplementary Figure 11. [file 41598_2022_26767_MOESM11_ESM.pdf]

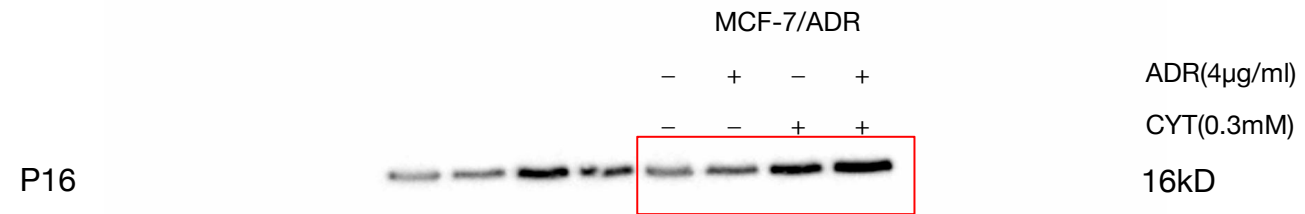

Supplementary Figure 12: Original for Figure 2(g).

Supplement: Supplementary file 12 — Supplementary Figure 12. [file 41598_2022_26767_MOESM12_ESM.pdf]

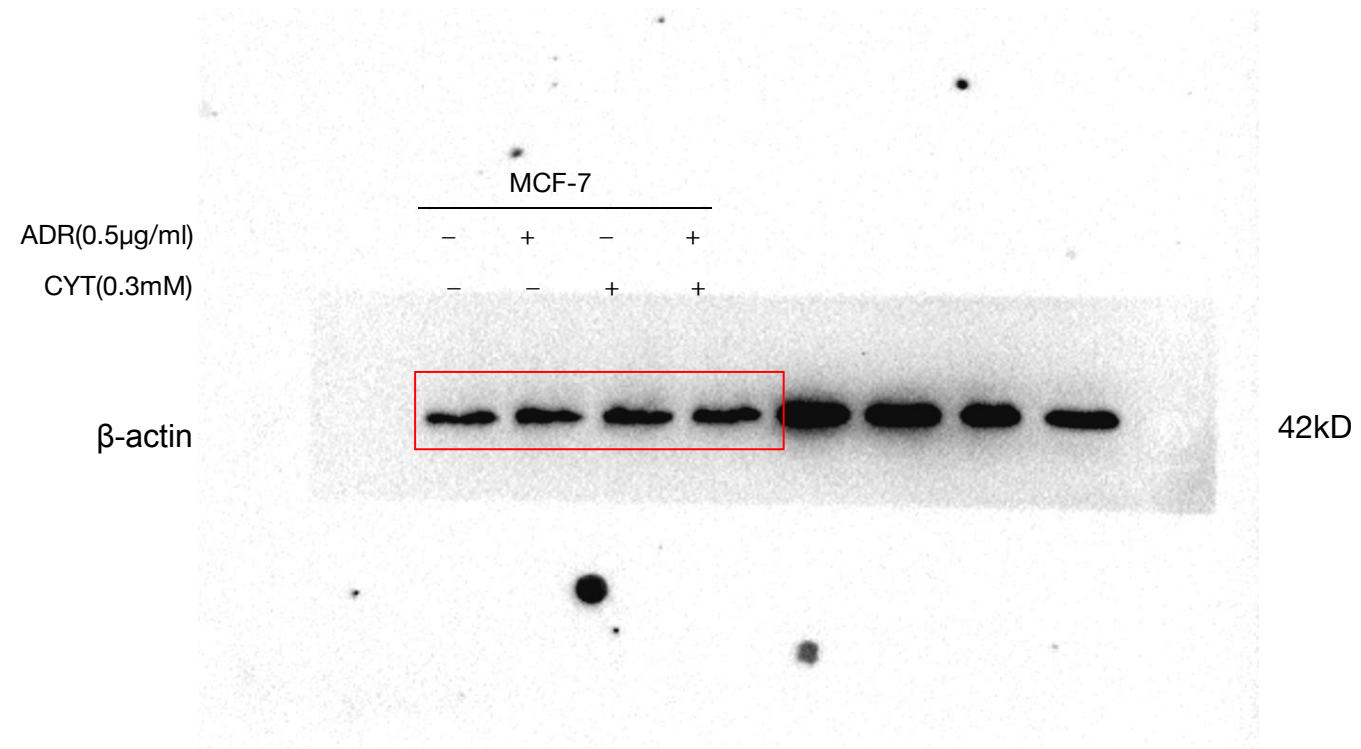

Supplementary Figure 13: Original for Figure 2(g).

Supplement: Supplementary file 13 — Supplementary Figure 13. [file 41598_2022_26767_MOESM13_ESM.pdf]

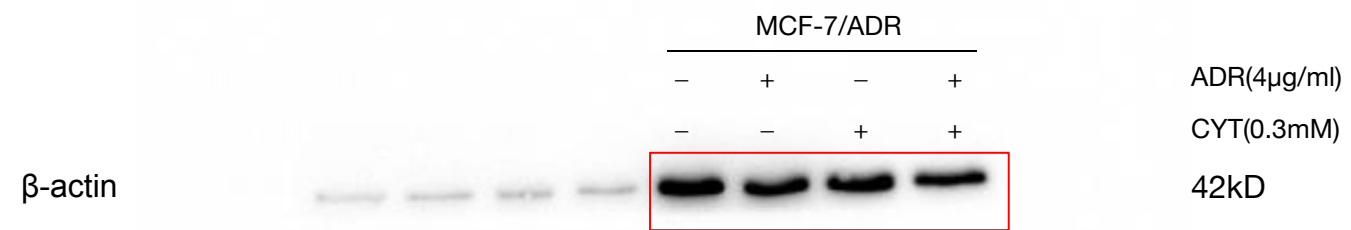

Supplementary Figure 14: Original for Figure 2(g).

Supplement: Supplementary file 14 — Supplementary Figure 14. [file 41598_2022_26767_MOESM14_ESM.pdf]

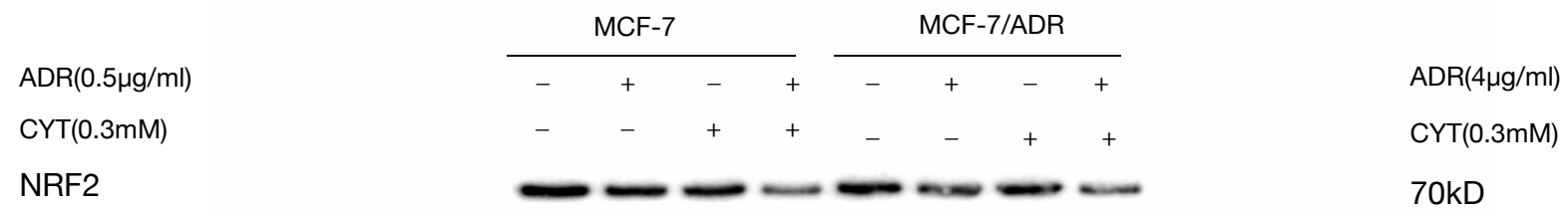

Supplementary Figure 15: Original for Figure 4(a).

Supplement: Supplementary file 15 — Supplementary Figure 15. [file 41598_2022_26767_MOESM15_ESM.pdf]

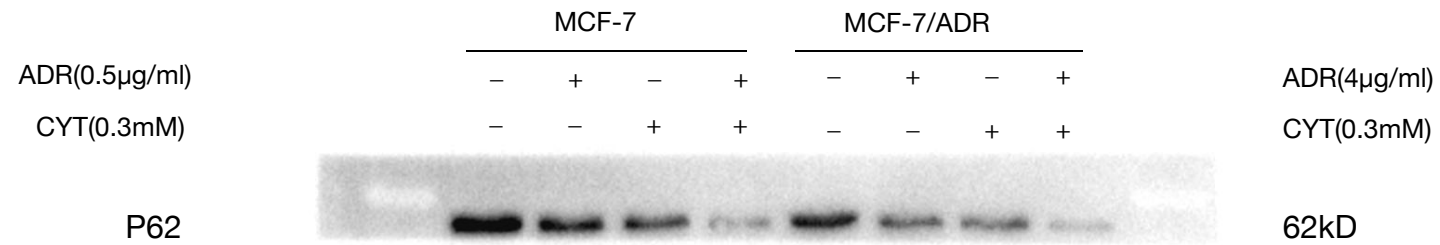

Supplementary Figure 16: Original for Figure 4(a).

Supplement: Supplementary file 16 — Supplementary Figure 16. [file 41598_2022_26767_MOESM16_ESM.pdf]

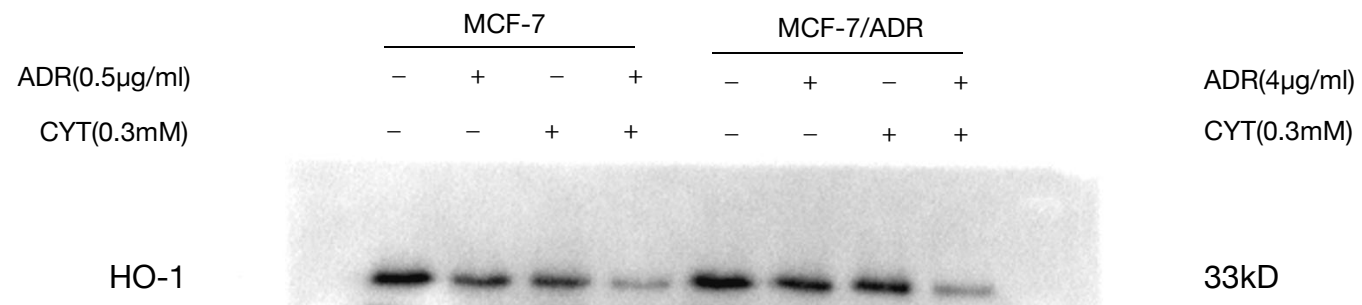

Supplementary Figure 17: Original for Figure 4(a).

Supplement: Supplementary file 17 — Supplementary Figure 17. [file 41598_2022_26767_MOESM17_ESM.pdf]

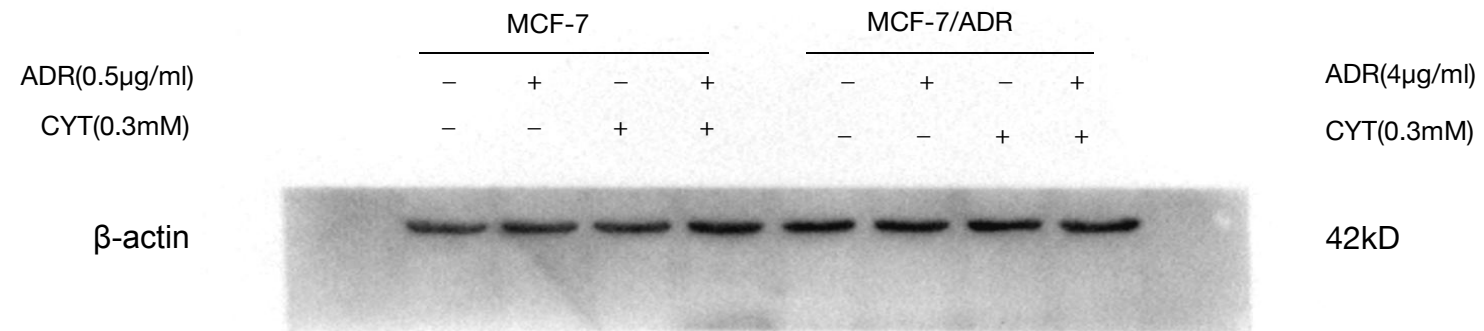

Supplementary Figure 18: Original for Figure 4(a).

Supplement: Supplementary file 18 — Supplementary Figure 18. [file 41598_2022_26767_MOESM18_ESM.pdf]
